# The proteomic investigation reveals interaction of mdig protein with the machinery of DNA double-strand break repair

**DOI:** 10.18632/oncotarget.4961

**Published:** 2015-07-22

**Authors:** Wei Wang, Yongju Lu, Paul M. Stemmer, Xiangmin Zhang, Yongyi Bi, Zhengping Yi, Fei Chen

**Affiliations:** ^1^ Department of Pharmaceutical Sciences, Eugene Applebaum College of Pharmacy and Health Sciences, Wayne State University, Detroit, MI, USA; ^2^ School of Public Health, Wuhan University, Wuhan, Hubei, P.R. China; ^3^ The Proteomics Core and Institute of Environmental Health Sciences, School of Medicine, Wayne State University, Detroit, MI, USA

**Keywords:** mdig, DNA repair, NHEJ, XRCC5, XRCC6

## Abstract

To investigate how mineral dust-induced gene (mdig, also named as mina53, MINA, or NO52) promotes carcinogenesis through inducing active chromatin, we performed proteomics analyses for the interacting proteins that were co-immunoprecipitated by anti-mdig antibody from either the lung cancer cell line A549 cells or the human bronchial epithelial cell line BEAS-2B cells. On SDS-PAGE gels, three to five unique protein bands were consistently observed in the complexes pulled-down by mdig antibody, but not the control IgG. In addition to the mdig protein, several DNA repair or chromatin binding proteins, including XRCC5, XRCC6, RBBP4, CBX8, PRMT5, and TDRD, were identified in the complexes by the proteomics analyses using both Orbitrap Fusion and Orbitrap XL nanoESI-MS/MS in four independent experiments. The interaction of mdig with some of these proteins was further validated by co-immunoprecipitation using antibodies against mdig and its partner proteins, respectively. These data, thus, provide evidence suggesting that mdig accomplishes its functions on chromatin, DNA repair and cell growth through interacting with the partner proteins.

## INTRODUCTION

Mineral dust-induced gene (mdig) was first identified in alveolar macrophages (AMs) isolated from coal miners who were exposed to mineral dust by working in the mining industry and had developed chronic lung diseases [1, 2]. Mdig was also independently identified in human glioblastoma cell line T98G cells with c-Myc overexpression and named as myc-induced nuclear antigen 53 (mina53) [3]. Because of the predominant localization of the gene product in the nucleoli of the cells, this gene was designated as nucleolar protein 52 (NO52) also [4]. A number of human cancers, including lung cancer [2], colon cancer [5], esophageal squamous cell carcinoma [6], gingival squamous cell carcinoma [7], lymphoma [8], neuroblastoma [9], gastric cancer [10], hepatocellular carcinoma [11], cholangiocarcinoma [12] and breast cancer [13], exhibited increased expression of the mdig mRNA or protein. More importantly, the expression level of mdig appears to be an important prognostic factor for some cancer patients, especially for those patients with earlier stages of adenocarcinoma lung cancer or breast cancer [13, 14]. In the cells with overexpression or silencing by siRNAs, recent experimental data suggest that mdig promotes cell proliferation but suppresses cell migration and invasion [15]. In a mouse lung fibrosis model, genetic disruption of the mdig gene ameliorates silica particle-induced lung fibrosis, possibly through reducing infiltrations of the macrophages and Th17 cells into the lung interstitium [16, 17].

Structurally, the mdig protein contains a conserved Jmj C domain that was commonly found in most of the classic histone demethylases [1, 3]. However, distinguishing from these known histone demethylases, the mdig protein lacks domains important for chromatin binding, such as WD repeats, PHD fingers and/or the Tudor domain. Previous reports suggested moderate effect of mdig on tri-methylation of histone H3 lysine 9 (H3K9me3) and the expression of ribosomal RNAs (rRNAs), H19, IGF2, Myc, Jhdm3a, and X56, a gene located in the heterochromatin regions [18], indicating that mdig protein may contribute to the epigenetic regulation and gene expression. Using peptide screening of mdig interactors in HEK293T cells, studies by Ge et al [19] revealed that mdig is able to hydroxylate histidine 39 of Rpl27a in the HXHR motif, suggesting that mdig may be a ribosomal oxygenase (ROX). Analysis of the crystal structural characteristics further demonstrated that mdig may serve as hydroxylase rather than the demethylase due to the presence of the C-terminal winged helix (WH) domain and the distinctive features of the Jmj C domain [20]. Thus, it is very likely that the influence of mdig on epigenetics is achieved through its interaction with other chromatin binding proteins.

Protein-protein interaction has long been viewed as the basis of physiological activities of any living cells, such as maintaining the cellular integrity, DNA replication, gene transcription, signal transduction, and immune response. Understanding the interaction partners of any given protein is essential for revealing the physiological or pathological mechanisms of human diseases [21]. In the past few years, proteomics approaches using high-performance liquid chromatography (HPLC)-tandem mass spectrometry (MS/MS) and co-immunoprecipitation (Co-IP) have been widely applied to identify key components of functional protein complexes [22, 23]. Many of the protein-protein interaction or so-called interactome studies, however, were made by overexpressing epitope-tagged bait proteins, which might alter the binding stoichiometry of the proteins investigated. To determine the authentic interaction partners of the mdig protein, we employed a straightforward, label-free approach combining HPLC-MS/MS with co-IP using human cancer cell line A549 cells and human bronchial epithelial cell line BEAS-2B cells, respectively [24]. This strategy allowed us to identify several proteins involved in the non-homologous end-joining (NHEJ) DNA repair and chromatin binding, including Ku80 (XRCC5), Ku70 (XRCC6), retinoblastoma-binding protein 4 (RBBP4), protein arginine methyltransferase 5 (PRMT5), etc., as the endogenous interaction partners of the mdig protein. Additional biochemical analyses indicated that interaction between mdig and XRCC5/6 sensitizes DNA double-strand break (DSB) induced by the radiomimetic drug, phleomycin. These data, thus, may shed new lights on our understanding of the oncogenic role of the mdig protein that was overexpressed in a number of human cancers.

## RESULTS

### Identification of mdig interaction proteins

**Figure 1:**
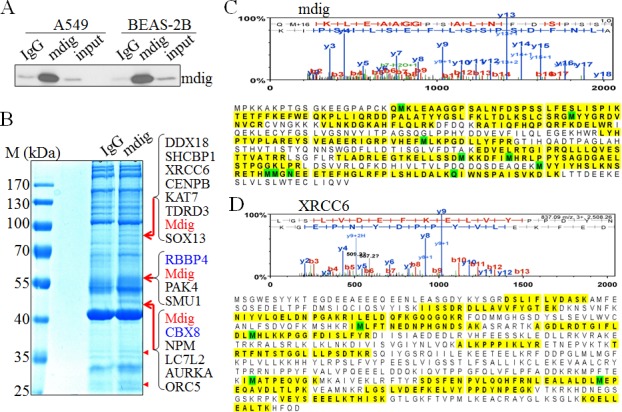
Proteomic investigation of the mdig-interacting proteins **A.** The immunoprecipitation (IP) efficiency of the polyclonal mdig antibody was tested in both A549 cells and the BEAS-2B cells. **B.** One-dimensional SDS-PAGE separation of the immunoprecipitated proteins using control IgG or mdig antibody from the A549 cells. Several protein bands that are unique in the mdig IP but not the IgG IP were marked by red arrows or red arrow heads. Proteins identified in these bands by MS/MS were listed on the right side of the panel. **C.** & **D.** Top panels show representative spectra of mdig **C.** and XRCC6 **D.**. The amino acids highlighted are peptides identified by MS/MS.

To enrich the naturally occurring mdig interaction proteins in the cells, we cultured A549 cells or BEAS-2B cells under the physiological condition without ectopic overexpression or metabolic labeling. The capability of the mdig antibodies in immunoprecipitation (IP) were first tested by incubating the cell lysates with both the polyclonal and monoclonal antibodies that were used in routine Western blotting experiments. Although both antibodies were able to pull-down the mdig protein, we found that the polyclonal antibody of mdig exhibited higher efficiency in IP (Figure 1A). To identify proteins that interact with mdig, the immunocomplexes from IP of the control IgG or mdig antibody was separated by SDS-PAGE gels, followed by staining with coomassie brilliant blue. As shown in Figure 1B, several unique protein bands were consistently presented in the complexes pulled-down by mdig antibody, but not in the control IgG in 4 independent experiments (pointed by arrows and arrowheads, Figure 1B). To determine the protein identities of these unique protein bands in the IP using mdig antibody, the three most clear-cut bands with molecular weight around 80, 60 and 45kDa (pointed by arrows) along with the gels at the same positions in the lane of IgG IP were excised, digested by trypsin and analyzed by Orbitrap Fusion™ Tribrid mass spectrometer (MS/MS). It was unexpected that mdig protein was detected in all of these bands, possibly due to alternatively spliced isoforms as we reported before or over-enrichment by IP and lagging in gel separation (Figures 1B and 1C). The other proteins in these bands include DDX18, XRCC6, CENPB, KAT7, TDRD3, RBBP4, ORC5, CBX8, NPM, etc. (Figures 1B, 1D and data not shown). XRCC6 (Ku70) is one of the key subunits of the non-homologous end joining (NHEJ) repair complex for the DNA double-strand break (DSB). RBBP4 and CBX8 had been previously demonstrated as important components of the Polycomb Repressive Complex 2 (PRC2) and PRC1, respectively. PRC2 silences transcription of the genes through inducing histone H3 lysine 27 trimethylation (H3K27me3). PRC1, on the other hand, can bind to H3K27me3 to catalyze H2A monoubiquitination. Thus, it is plausible to speculate that mdig may be involved in the regulation of DNA repair and epigenetic silencing by PRC2 and PRC1.

### Mdig is in complexes with XRCC6 or RBBP4

**Figure 2:**
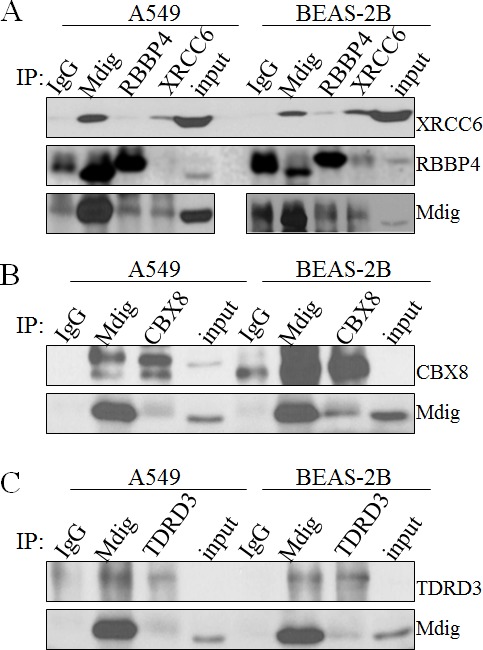
Validation of mdig interacting proteins **A.** Coimmunoprecipitation of endogenous mdig with the endogenous XRCC6 and RBBP4 from both A549 cells and BEAS-2B cells. **B.** Coimmunoprecipitation of endogenous mdig with the endogenous CBX8 from both A549 cells and BEAS-2B cells. **C.** Coimmunoprecipitation of endogenous mdig with the endogenous TDRD3 from both A549 cells and BEAS-2B cells.

To verify the results of MS/MS, we next examined the interaction of mdig with its partners through co-IP. Although more than 15 proteins were detected among the 3 major bands in mdig IP in MS/MS, we initially focused our attentions on XRCC6, RBBP4, TDRD3, and CBX8 because of their well-known function in DNA repair and chromatin modification. Endogenous mdig and/or its partner proteins were immunoprecipitated with the indicated antibodies followed by Western blotting using the corresponding antibodies. Indeed, in both A549 cells and BEAS-2B cells, we found XRCC6 and RBBP4 in the mdig IP (Figure 2A). Vice versa, mdig was found in the IPs of XRCC6 and RBBP4, respectively. However, no direct interaction between XRCC6 and RBBP4 was detected in A549 cells, suggesting that mdig might form different complexes with XRCC6 and RBBP4 in these cells. The antibody we used for RBBP4 has cross reactivity with RBBP7 (RbAP46) due to higher homologues in amino acid sequences between these two proteins, which might explain the subtle difference in protein migration position in Western blotting (middle panel, Figure 2A).

Co-IP experiments were also conducted to confirm the interaction of mdig with CBX8 and TDRD3, respectively. CBX8, a homolog of Drosphila Polycomb protein, was originally characterized as a central subunit of the PRC1 complex for transcriptional repression. As depicted in Figure 2B, we detected interaction between mdig and CBX8 in A549 cells as well as in BEAS-2B cells. A marginal interaction between mdig and TDRD3, a Tudor domain containing protein that was previously identified as a binding protein of the dimethylarginine on H3R17me2, was observed (Figure 2C).

### The expression levels of mdig affect DNA damage responses

**Figure 3:**
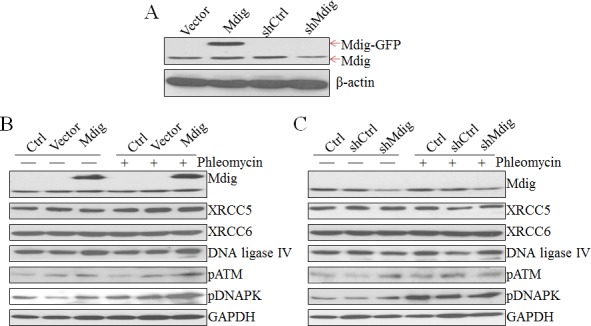
Regulation of the NHEJ repair signaling by mdig **A.** Immunoblotting shows overexpression of exogenous mdig-GFP protein and silencing of mdig by mdig shRNA in the A549 cells. **B.** Overexpression of mdig enhances phleomycin-induced activation of ATM and DNA-PK as evidenced by the increased levels of phospho-ATM (pATM) and pDNA-PK. **C.** Silencing mdig reduced phleomycin-induced activation of ATM and DNA-PK.

Considering the direct interaction of mdig with XRCC6, a molecular scaffold with a ring structure that recognizes the ends of double-strand breaks (Figures 1 and 2), we further explored the role of mdig on DNA DSB repair by NHEJ. We first established A549 cell lines with stable expression of control vector, mdig-GFP, control shRNA (shCtrl), or shMdig that silences mdig. Immunoblotting confirmed overexpression of mdig by transfection of mdig-GFP or silencing of the mdig protein by the expression of shMdig (Figure 3A). DSB were induced by the treatment of the cells with 30 μM phleomycin for 30 min. Overexpression of mdig did not influence the protein levels of XRCC5, XRCC6, or DNA ligase IV. A modest increase of phosphorylated ATM (pATM) and phosphorylated DNA-PK (pDNA-PK) was noted in the cells expressing mdig-GFP and treated with phleomycin (Figure 3B). The effect of mdig on pATM and pDNA-PK was additionally confirmed in the cells expressing mdig-targeting shRNA. Silencing mdig by shMdig minimized induction of pATM and pDNA-PK by phleomycin (Figure 3C). These data, thus, suggest that mdig may enhance the DNA damage responses (DDR) of the cells treated with phleomycin.

### Mdig sensitizes phleomycin-induced DNA double-strand breaks

**Figure 4:**
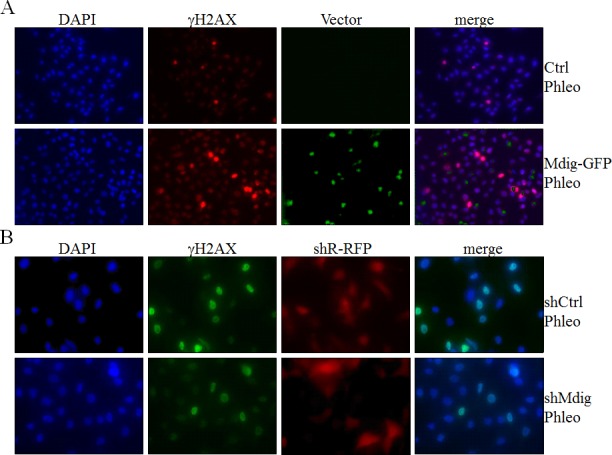

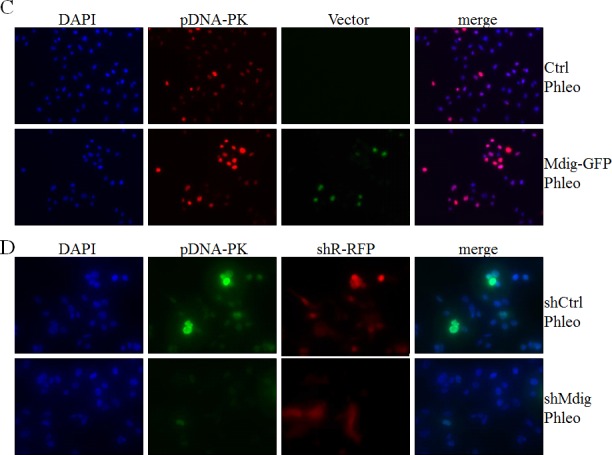

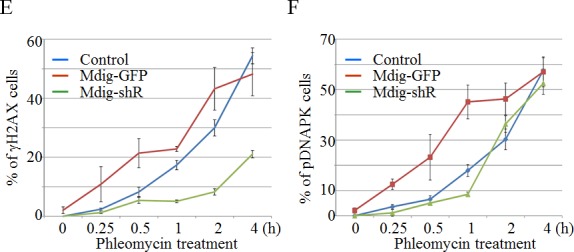
Mdig enhances DSB in response to phleomycin (Phleo) **A.** Immunofluorescent staining of the DSB indicator, γH2AX. The stably transfected A549 cells with control vector (Ctrl) or mdig-GFP were treated with phleomycin for 1h followed by DAPI and γH2AX staining. **B.** The stably transfected A549 cells with control shRNA (shCtrl) or mdig shRNA (shMdig) were treated with phleomycin for 1h followed by DAPI and γH2AX staining. **C.** Immunofluorescent staining of the DSB indicator, pDNA-PK. The stably transfected A549 cells with control vector (Ctrl) or mdig-GFP were treated with phleomycin for 1h followed by DAPI and pDNA-PK staining. **D.** The stably transfected A549 cells with control shRNA (shCtrl) or mdig shRNA (shMdig) were treated with phleomycin for 1h followed by DAPI and pDNA-PK staining. **E.** Time course of the phleomycin-induced γH2AX signal in control cells or the cells expressing mdig-GFP or mdig shRNA. **F.** Time course of the phleomycin-induced pDNA-PK signal in control cells or the cells expressing mdig-GFP or mdig shRNA.

Above data revealed physical interaction of mdig with XRCC6 and its involvement in the activation of two key DNA damage response kinases, ATM and DNA-PK, in the cells treated with phleomycin that induces DSB of the DNA. An early marker of the DNA DSB is a phosphoepitope that appears on serine 139 of the H2A variant, H2AX, to form γH2AX in chromatin around the breakage sites. The level and perpetuation of γH2AX are not only indicators of DNA DSB but also suggestive of the repairing capacity of the cells on the damaged DNA. To determine whether interaction between mdig and XRCC6 affects the NHEJ repair, we measured the level of γH2AX by immunofluorescent staining following phleomycin treatment of the cells with overexpression or silencing of the mdig protein. As shown in Figure 4A, cells expressing a control vector exhibited marginal increase of γH2AX in response to phleomycin (top panels, Figure 4A). A substantial increase of the γH2AX positive cells was noted in cells with an overexpression of mdig and treated with phleomycin (bottom panels, Figure 4A). Silencing mdig by the expression of the mdig-targeting shRNA (shMdig), on the other hand, reduced the level of γH2AX induced by phleomycin (bottom panels, Figure 4B). Similar patterns of the pDNA-PK were observed in these cells with overexpression or silencing of the mdig (Figures 4C & 4D).

To better delineate the effect of mdig on NHEJ repair, we performed a kinetic analysis of the DNA damage response by studying the time courses of γH2AX and pDNA-PK in these cells described above. Immunofluorescent staining revealed that about 10% of the mdig-overexpressing cells were γH2AX positive at 15 min after phleomycin treatment. At 30 min to 1 h, more than 20% of these cells were γH2AX positive. In contrast, the frequencies of γH2AX positive cells remained much lower in the cells in which mdig was silenced by shRNA (Figure 4E). The patterns of pDNA-PK roughly resemble the time-dependent changes of the γH2AX in both the mdig overexpressing and silencing cells (Figure 4F).

### Mdig interacts with multiple functional protein modules

**Figure 5:**
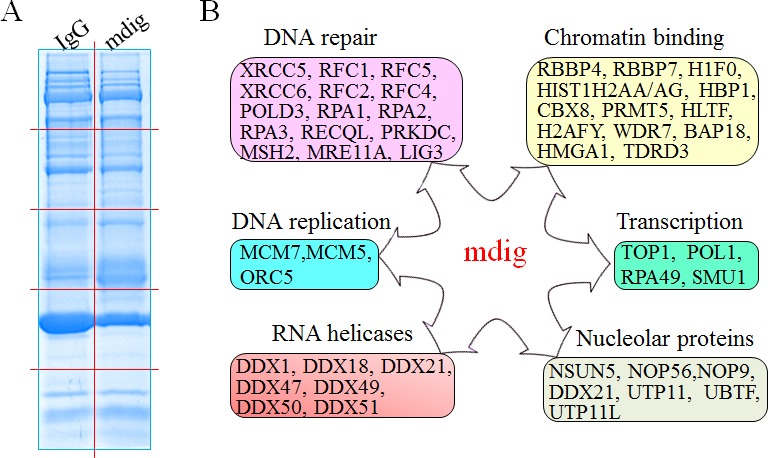
Functional pathways of mdig-interacting proteins **A.** Separation of the IP products of IgG IP and mdig IP from the A549 cells by 1D SDS-PAGE. The gel was stained by Coomassie blue staining. Red lines indicate cuts of gel slices subjected to proteomic analysis. **B.** Schematic overview of the functional groups of proteins that interact with mdig as identified by proteomic analyses.

It was very likely that many low-abundant proteins that interact with mdig in IP couldn’t be visualized as individual bands in the gel by coomassie blue staining. To explore additional interaction proteins of mdig, the IP products of mdig or IgG were separated by one dimensional SDS-PAGE, and the whole lanes of the gel were randomly sliced and subjected to proteomic analysis to maximize the coverage of the mdig-interacting proteins (Figure 5A). To reduce false positives caused by nonspecific binding during IP, we prepared IP samples in 3 independent experiments, one of which had 4 replications, and analyzed in two laboratories using different MS/MS systems. The resulting set of 207 proteins includes not only the XRCC6, RBBP4, TDRD3, and others that we had detected in the first experiment (Figure 1), but also additional proteins involved in DNA repair, chromatin binding, RNA processing, gene transcription, DNA replication, and nucleolar function (Figure 5B). For those proteins that are important for DNA damage response and repair, several proteins that are known to contribute to DNA NHEJ repair were identified as mdig-interacting proteins, including XRCC5, XRCC6, DNA-PK (PRKDC), RFC1, RFC2, POLD3, etc.. Meanwhile, some of the proteins identified as mdig-interacting proteins had previously been shown as chromatin-binding proteins for epigenetic regulation. For example, RBBP7, a protein usually found associated with RBBP4 in the Polycomb Repressive Complex 2 (PRC2) that binds to chromatin to catalyze the formation of the repressive chromatin marker, H3K27me3. It is not unexpected to identify some mdig-interacting proteins as nucleolar proteins or RNA helicases, given the fact that mdig is mostly localized in the nucleoli and involved in the biogenesis of rRNAs. Interestingly, one of the mdig-interacting RNA helicases, DDX1, had been previously shown as an important accessory protein that facilitates repairing of the double-strand DNA break by clearing RNAs at the damage sites [25].

### Identifying mdig-interacting proteins in other type of the cells

**Figure 6:**
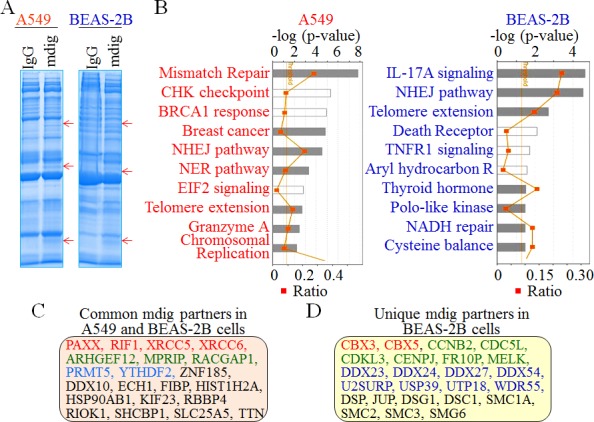
Comparison of the mdig-interacting proteins between A549 cells and the BEAS-2B cells **A.** Immunocomplexes of IgG IP and mdig IP from both A549 cells and BEAS-2B cells were separated by 1D SDS-PAGE. Red arrows indicate unique protein bands in mdig IPs commonly found in both A549 cells and BEAS-2B cells. **B.** Top 10 pathways of the mdig-interacting proteins in A549 cells (left) and BEAS-2B cells as identified by MS/MS. **C.** Selected common proteins identified in A549 cells and BEAS-2B cells that interact with mdig. Red: DNA repair protein; green: Rho GTPase pathway; blue: proteins involved in protein or RNA methylation; black: proteins in other functions. **D.** Selected mdig-interacting proteins that were detected in BEAS-2B cells but not the A549 cells. Red: H3K9me3 binding proteins; green: cell growth regulatory proteins; blue: proteins contribute to RNA processing; black: proteins in other functions.

It has been well known that protein-protein interaction may differ considerably among a wide spectrum of cell types due to the varied abundance of the individual proteins. To investigate whether some of these mdig-interacting proteins identified above are ubiquitous or cell type specific, we also conducted mdig IP and proteomic analysis using BEAS-2B cells and compared the interacting proteins with those found in A549 cells. The banding pattern obtained with SDS-PAGE is very similar for the BEAS-2B and A549 cell mdig IP samples. Each mdig IP lane contains three clear bands that are absent from the control IgG IP (Figure 6A, indicated by arrows). Ingenuity Pathway Analysis (IPA) following the proteomic profiling of the mdig-interacting proteins in these two different cell types revealed a similar enrichment of the proteins in the pathways of DNA NHEJ repair and telomere extension (Figure 6B). However, some pathways are unique in the BEAS-2B cells, such as signaling pathways of the IL-17A, TNFR1, and aryl hydrocarbon receptor. Detailed analysis of the proteins that are isolated with mdig IP suggested that in both A549 cells and BEAS-2B cells, mdig can interact with the DNA repair proteins, including XRCC5, XRCC6, PAXX, RIF1, and the arginine methyltransferase 5 (PRMT5), histone H2A (HIST1H2A), Rho guanine nucleotide exchange factor 12 (ARHGEF12), etc. (Figure 6C). Notably, several proteins that interact with mdig were detected only in the BEAS-2B cells (Figure 6D), such as CBX3 (HP1γ), CBX5 (HP1α), CCNB2, CDC5L, CDKL3, etc.. In addition to their long-established role in maintaining the heterochromatin states by binding to H3K9me3, the CBX family proteins had been shown to be capable of binding to the lysine-methylated DNA-PK and XRCC5 to localize the DNA repair machinery to DSB sites [26-28].

## DISCUSSION

In mammalian cells, genomic DNA is constantly damaged, even in normal homeostasis. A plethora of environmental factors, such UV radiation, chemicals and heavy metals, can trigger severe DNA damage [29]. To prevent accumulative mutations due to DNA damage and the consequent tumorigenesis, the cells deploy a number of DNA repair machineries to maintain the stability and integrity of the genome. These repair machineries, collectively known as the DNA damage response (DDR), involve detection of the DNA lesion, initiation and propaganda of the damage signal, and establishment of conditions that ensure that DNA repair is started and completed [30]. In addition to cell cycle arrest and transcriptional termination at the DNA damage sites, proper chromatin states, including chromatin remodeling, nucleosome rearrangement, and histone methylation and ubiquitination, provide the necessary platform to coordinate the DDR.

We and others had identified mdig gene from the human alveolar macrophages and tumor cell lines, respectively [1, 3, 4]. The mdig protein contains a Jmj C domain that is viewed as a signature motif of a large family of histone demethylases that regulate the chromatin status during DNA replication and gene transcription. However, mdig protein lacks critical domains for chromatin binding, such as WD repeats, PHD fingers and/or the Tudor domain. Despite overexpression or silencing experiments in certain type of cells suggested involvement of mdig on the methylation states of the histone H3 protein and the expression of rRNA, H19, IGF2, Myc, Jhdm3a, and X56 [2, 18], it remains to be fully elucidated whether mdig exhibits its regulatory role on chromatin or gene expression is achieved through interaction with other chromatin binding proteins. In the present report, we employed a strategy of IP in combination with protein profiling by proteomic analysis to investigate the endogenous proteins that interact with mdig. Our data indicate that mdig physically interacts with the NHEJ repair complex containing XRCC5, XRCC6 and DNA-PK in response to DNA DSB. This interaction sensitizes phleomycin-induced double-strand breaks (DSB) of the DNA in A549 lung cancer cells. Silencing mdig by shRNA, on the other hand, alleviated DNA DSB induced by phleomycin (Figure 4). In addition to the verification in four independent experiments using two different MS/MS systems, the interaction of mdig with XRCC5 and XRCC6 was also confirmed in two other cell types, the human bronchial epithelial cell line BEAS-2B (Figure 6) and human multiple myeloma cell line H929 (Wu et al, unpublished observations). Taken together, these results suggested a previously uncharacterized role of mdig, the participation in NHEJ repair processes of the DNA DSB. Association of mdig with the complex of XRCC5, XRCC6 and DNA-PK may weaken the NHEJ repair capacity (Figures 4E & 4F) and consequently, increase the opportunity of genomic mutation and carcinogenesis in response to toxins that target genomic DNA.

In addition to the NHEJ repair machinery, both proteomic analysis and co-IP experiment also unraveled interaction of mdig with RBBP4 and CBX8, the key subunits of the PRC2 and PRC1, respectively. Both PRC2 and PRC1 had been traditionally viewed as epigenetic regulators to promote silent chromatin through H3K27me3 and the ubiquitination of the histone proteins [31]. Recent studies also revealed rapid recruitment of these two complexes to the sites of DNA DSB for NHEJ repair [32]. Functional disruption of either PRC2 or PRC1 impaired the DNA repair process or sensitized DNA to damage following ionizing radiation (IR)-induced DSB [33, 34]. However, it is unclear at the present whether our findings that mdig interacts with RBBP4 and CBX8 are suggestive of mdig’s interaction with PRC2 or PRC1 directly at the DNA damage sites, despite our co-IP experiments detected weak association between mdig and the EZH2 protein, the enzymatic component of the PRC2 complex (data not shown). Thus, the possibility that these interactions may be beyond the PRC2 and PRC1 cannot be ruled out. Indeed, several lines of evidence implied PRC2 independent role of RBBP4. RBBP4 has long been identified as an essential subunit of the nucleosome remodeling and deacetylase (NuRD) complex that regulates chromatin structure, gene transcription, DNA damage repair, and aging [35]. Meanwhile, RBBP4 may also be involved in the activity of chromatin assembly factor-1 (CAF-1), a complex acting as histone chaperone for assembly of nucleosome, restoration of chromatin after DNA damage and heterochromatin duplication [36]. Furthermore, it is especially interesting to note that RBBP4 has been linked to the human aging process with hippocampus-related memory loss [37]. Inhibition of RBBP4 in the forebrain of young mice caused a reduction of the HAT activity of CBP on the acetylation of histone H4 lysine12 and histone H2B lysine20, leading to memory impairment. Our gene knockout studies showed that mice with mdig gene deficiency live longer than their wild counterpart [13], possibly suggesting an inhibitory role of mdig on RBBP4 through direct interaction. Partial deletion of mdig gene might indirectly enhance the function of RBBP4 and postpone the onset of memory loss and other aging processes.

It is not surprising to detect interaction of mdig and the CBX3 (HP1γ) and CBX5 (HP1α) proteins in the BEAS-2B cells. Both CBX3 and CBX5 are H3K9me3-binding proteins for the formation of the heterochromatin and the maintenance of the genomic stability. Our previous studies in BEAS-2B cells implicated possible involvement of mdig in the demethylation of H3K9me3 [2, 18]. Thus, at the global level, such an interaction of mdig and CBXs may contribute to the balance between euchromatin and heterochromatin through fine-tuning the binding capability of CBXs to H3K9me3. At the DNA damage sites, this interaction may impede the function of CBX proteins in maintaining suitable chromatin states for DNA repair and the interaction of CBX proteins with other DNA repair proteins. A number of studies have demonstrated involvement of CBX proteins in DNA repair [38]. CBXs may recruit BRCA1/BARD1 to the DNA damage sites through direct binding to BRCA1/BARD1 and the H3K9me3 during homologous recombination (HR) repair of the DSB [39]. Although the role of CBXs in NHEJ repair is unclear, they may contribute to the restoration of the correct chromatin states when the damages are repaired. However, it should be noted that interaction of mdig with CBX3 and CBX5 may be cell type specific, because such an interaction was only observed in BEAS-2B cells but not in A549 cells or the H199 cells.

There are a number of other proteins that were identified as mdig-interacting proteins, such as RNA helicases and several nucleolar proteins. It is unclear whether interaction of mdig with these proteins is also involved in DDR or other independent functions. An additional interesting protein that was identified as an interacting protein of mdig in all three different cell lines is the protein arginine methyltransferase 5 (PRMT5) that specializes in arginine methylation of both histones and non-histone proteins that are involved in cell cycle arrest, apoptosis and DNA repair [40]. This interaction between mdig and PRMT5 may explain the regulatory roles of mdig on the CD4^+^ T cells [41], Th2 bias [42] and Th17 cells [16, 17]. It has been shown that PRMT5-catalyzed symmetrical dimethylation of H4R3 is the most repressive histone marker for the global expression of the genes in CD4^+^ T cells [41, 43]. It is also known that arginine methylation augments the transcriptional ability of NIP45 on Th2 cytokine IL-4 [44, 45]. Binding of PRMT5 by mdig, therefore, may antagonize the function of PRMT5, leading to repression of IL-4 secretion and Th2 cell differentiation. For the differentiation of the Th17 cells, binding of mdig to PRMT5, a known JAK-binding protein, may alter the dynamics of the JAK-STAT3 signaling that is essential for the specialization of the Th17 cells [46].

Together, our results have unraveled previously unknown functions of mdig in interacting with the proteins in DDR and possibly other important cellular processes. These interactions reinforced the regulatory role of mdig on epigenetics and chromatin organization, and supported the idea that mdig can be recruited to specific chromatin sites through association with its partner proteins that are able to establish and/or recognize unique epigenetic markers on the chromatin. By extension, our findings may help to redefine the structure and enzymatic activities of the mdig protein, considering the fact that active interactions with other proteins may alter the positions of the key domains that influence the substrate accessibility and the enzymatic activities. Several questions, however, remain to be answered. For example, previous studies using peptide screening strategy demonstrated association of mdig with the ribosomal protein Rpl27a and the subsequent histidine 39 hydroxylation of the Rpl27a [19, 20, 47]. Does interaction of mdig with these NHEJ repair proteins and others as reported here also induce hydroxylation and alter the activity/function of these proteins? It’ll be also interesting to know why Rpl27a was not discovered as an interacting protein of mdig in the current study using three different cell lines. One possibility is the limited sensitivity of IP experiment to pull down the endogenous proteins that associated with mdig. Additional tests are needed to answer these questions, define the domains in the mdig proteins that interact with other proteins and learn the detailed role of mdig in DNA repair, chromatin organization and tumorigenesis.

## MATERIALS AND METHODS

### Cell culture and transfection

The human lung carcinoma cell line A549 and bronchial epithelial cell line BEAS-2B were purchased from the American Type Culture Collection (ATCC, Manassas, VA). The cells were cultured in RPMI-1640 or DMEM medium (Invitrogen, Grand Island, NY) with 10% fetal bovine serum (Invitrogen, Grand Island, NY), 1% penicillin-streptomycin (Sigma, St. Louis, MO) and 1% L-Glutamine, and maintained in humidified incubator at 37°C with 5% CO2. For cell transfection, a total of 5 × 10^5^ A549 cells per well were seeded into 6-well plates until they reached 60-70% confluency. Transfections were performed using Lipofectamine 2000 (Invitrogen, Grand Island, NY) with mdig-GFP expression vector, control vector, RFP-conjugated mdig shRNA, and RFP-conjugated control shRNA (Origene, Rockville, MD). Stably transfected clone were established by the addition of G-418 (500 μg/ml) or puromycin (2 μg/ml) (Invitrogen, Grand Island, NY), and selected under fluorescent microscopy.

Co-immunoprecipitation—Cells were washed with phosphate buffered saline (PBS), followed by constantly agitating in non-denaturing lysis buffer (20 mM Tris-HCl pH 7.5, 137 mM NaCl, 10% glycerol, 1% Nonidet P-40, 2 mM EDTA, and 1 × protease inhibitor cocktail) for 30 min at 4°C. The proteins in the supernatant were collected by centrifuging at 12,500 rpm for 15 min at 4°C. For co-immunoprecipitation experiments, pre-cleared protein extracts (4 μg proteins for proteomics tests, and 400 μg proteins for immunoblot experiment) were incubated with Protein A/G PLUS-Agarose (Santa Cruz Biotechnology) coupled with rabbit polyclonal antibody against mdig, mouse monoclonal antibodies against XRCC6 and RbAp48 (RBBP4) (Abcam, Cambridge, MA), rabbit polyclonal antibodies against CBX8, TDRD3, and HBO (Bethyl Laboratories, Montgomery, TX), normal rabbit IgG or normal mouse IgG (Santa Cruz Biotechnology) at 4°C overnight, and the immunocomplexes were eluted by boiling with 2 × SDS-sample buffer after three washes with lysis buffer. Proteins were resolved by 10% polyacrylamide Tris-glycine SDS gels and stained by coomassie brilliant blue.

### Peptides preparation and Mass spectrometry

Specific gel bands in mdig lanes and regions from IgG lanes at the same position were excised from the gel. The gel pieces were first washed with water and 25 mM NH_4_HCO_3_, 50% ACN for 15 min each, and then dehydrated in 100% ACN for 5 min. After rehydrating in 50 mM NH_4_HCO_3_, and dehydrating in 100% ACN, the gel pieces were speed vac’ed dry for 5 min. Proteins were reduced in 5 mM DTT, 50 mM NH_4_HCO_3_; alkylation with 15 mM IAA, 50 mM NH_4_HCO_3_; and overnight digestion with sequencing-grade trypsin (Promega) in 25 mM NH_4_HCO_3_, 10% ACN. Following digestion, peptides were extracted from the gel using 50% ACN, 0.05% FA. The free peptides were then speed vac’ed to dryness and solubilized in 2% ACN, 0.1% FA. The peptides were separated by reverse phase chromatography (Acclaim PepMap100 C18 column, Thermo Scientific), followed by ionization with the Nanospray Flex Ion Source (Thermo Scientific), and introduced into an Orbitrap Fusion™ Tribrid mass spectrometer (Thermo Scientific). Alternatively, the peptides were separated and analyzed through Orbitrap XL.

### Proteome analysis

Abundant species were fragmented with high energy collision-induced dissociation (HCD). Data analysis was performed using Proteome Discoverer 1.4 (Thermo) which incorporated the Mascot algorithm (Matrix Science). The SwissProt_2014_03 database was searched for human protein sequences and a reverse decoy protein database was run simultaneously for false discovery rate (FDR) determination. Secondary analysis was performed using Scaffold 4.2.1 (Proteome Software). Minimum protein identification probability was set at ≥ 99% with 2 unique peptides at ≥ 99% minimum peptide identification probability. The specific mdig-interacting proteins were determined based on the criteria that the peptides were detected in three out of four experiments with a Mascot score higher than 50 and at least a 3-fold higher than the corresponding control immunoprecipitation. The interaction map between detected proteins was formed by Ingenuity Pathway Analysis (IPA).

### Immunoblots

Some of the mdig-interacting proteins were validated by co immunoprecipitation (Co-IP). Protein samples were pulled down as introduced in co-IP method from the mdig overexpression and mdig knockdown cells that were treated by 30 μm/ml phleomycin for 30 min, and lysed by RIPA buffer (Millipore, Billerica, MA) supplemented with phosphatase/protease inhibitor cocktail and 1 mM PMSF through sonication and centrifugation. The proteins were quantified by Micro BCA Protein Assay Reagent Kit (Thermo Scientific, Pittsburgh, PA). After boiled in LDS sample buffer (Invitrogen) containing 1 mM dithiothreitol, proteins were separated by 10% SDS-PAGE gels, and transferred onto PVDF membranes (Invitrogen). Blocked in 5% non-fat milk/Tris-buffered saline with 0.05% Tween-20 (TBS-T) at room temperature for 1 h, the membrane were incubated sequentially with primary and the second antibody, followed by image development using ECL substrates (Thermo Scientific, Pittsburgh, PA/ Millipore, Billerica, MA) and X-ray film. The primary antibodies include the mouse monoclonal antibody against mdig (MINA53) (Invitrogen), Rabbit monoclonal antibodies against Ku70, RbAp46/48 (Cell Signaling Technology), CBX8, TDRD3, HBO (Bethyl Laboratories, Montgomery, TX), Non-Homologous End Joining Panel (H2A.X, gamma H2A.X pS139, DNA Ligase IV, Ku70, Ku80), DNA Damage Kinases Panel (ATM, ATM phospho S1981, DNA-PKcs, DNA-PKcs phospho S2056) (Abcam, Cambridge, MA). The second antibodies include mouse monoclonal SB62a Anti-Rabbit IgG light chain (HRP) and Rat monoclonal H139-52.1 Anti-Mouse kappa light chain (HRP).

### Immunofluorescence

Cells were seeded (4 × 10^4^ cells/well) in a 24-well plate with 12mm circle cover glassed (Thermo Fisher Scientific) in 1 ml medium per well and cultured for 24 h. Then the cells were treated with phleomycin (Sigma-Aldrich) (30 μM/ml) for 0 min, 15 min, 30 min, 1 h, 2 h, or 4 h. The cells were then washed with PBS twice, fixed with 4% formaldehyde in PBS for 15 min, and permeabilized with 0.1% Triton X-100 in PBS solution for 10 min at room temperature. After washing with PBS 3 times, the cells were incubated with blocking buffer (PBS containing 10% goat serum, 1% BSA and 0.1% tween) for 1 h at room temperature. The cells were then incubated in phospho-H2AX or phospho-DNA-PKCs primer antibody (Abcam, Cambridge, MA) (5 μg/ml in PBS containing 1% BSA and 0.1% tween) overnight at 4°C, and the fluorochrome-conjugated second antibody (5 μg/ml in PBS containing 1% BSA and 0.1% tween) for 1 h at room temperature, followed by rinsing in PBS 3 times for 5 min each. The slides were mounted with a small drop of ProLong^®^ Gold Antifade Mountant with DAPI (Thermo Scientific, Pittsburgh, PA) and viewed under a fluorescence microscope.

### Statistics

The statistical significances were determined by Microsoft Excel. The data are expressed as mean ± standard deviation (SD). The student’s *t*-tests were used to determine the statistical significance of differences between samples treated under different conditions. Differences were considered statistically significant when *p* < 0.05.
